# The relationship between mental health literacy and psychological support-seeking attitudes in Syrian immigrant students

**DOI:** 10.1186/s12889-025-23816-8

**Published:** 2025-09-30

**Authors:** Havva Kaçan, Vasfiye Bayram Değer, Sema Çi̇fci̇

**Affiliations:** 1 Department of Nursing, Faculty of Health Sciences, Kastamonu University, Kastamonu, Türkiye; 2 Faculty of Medicine/Department of Public Health, Mardin Artuklu University, Mardin, Türkiye; 3 Faculty of Health Sciences/Public Health Nursing, Mardin Artuklu University, Mardin, Turkey

**Keywords:** Mental health literacy, Seeking psychological support, Immigrant students

## Abstract

**Background:**

The study was conducted to examine the relationship between mental health literacy and psychological support-seeking attitudes among Syrian immigrant university students.

**Materials and methods:**

The population of the study consisted of 2000 Syrian immigrant students studying in different departments of a university, and the sample comprised 326 immigrant students. In the study, data were collected through Descriptive Questions, the Mental Health Literacy Scale (MHLS), and the Attitude Scale toward Receiving Professional Psychological Support-Short Form (ATSPPH-SF).

**Results:**

The mean MHLS score of Syrian immigrant students was 11.804 ± 3.434. The average score of the ATSPPH-SF was 58.245 ± 8.131. A positive and moderate correlation was found between the total score of MHL and psychological support (*r* = 0.514, *p* < 0.01). The rate of explaining the effect of MHL on receiving psychological support was found as 26.2%. It was found that the increase in the level of knowledge positively affected the attitudes of the students to seek psychological support, and the belief-oriented dimension had a significant and positive effect on the attitude toward psychological support (B = 0.918, β = 0.250, *p* < 0.001). A one-unit increase in the belief-oriented dimension provided a 0.918-unit increase in the psychological support attitude score. However, it was observed that the effect of this dimension was lower compared with the information-oriented dimension. This finding shows that belief-based approaches may positively affect the attitude toward psychological support, but their effect is limited.

**Conclusions:**

It was determined that the MHL of the students was moderate and the attitudes of seeking psychological support were positive. There was a significant and positive relationship between the mental health literacy of Syrian immigrant students and seeking psychological support. It can be argued that initiatives to increase MHL can positively shape individuals’ attitudes toward psychological support.

**Supplementary Information:**

The online version contains supplementary material available at 10.1186/s12889-025-23816-8.

## Introduction

Increasing mental illnesses in the world and our country affect emotions, thoughts, mental state, communication ability, and daily life, and they also make it difficult for people to perform their social and professional activities due to the economic and emotional challenges and restrictions they cause [1, 2]. Therefore, it has become a common goal to maximize the quality of life of people with mental illness around the world and to ensure their participation in economic and social life. In achieving these goals, one of the most important strategies to reduce the burden of mental illnesses is stated as the development of mental health literacy (MHL)” [3], which is defined as knowledge and beliefs related to recognizing, managing, and preventing mental disorders [4]. Considering these definitions, the purpose was to increase MHL and to recognize people’s mental disorders at an early stage, seek professional help, and restore people’s health with appropriate care and treatments [5].

Considering that mental disorders occur in adolescents and young adults at a rate of 70–75% [6, 7], it can be argued that we are a risky country in terms of mental disorders. At this point, it is even more important to increase the MHL of young people, who will be the future adults of society, to MHL more broadly. Teaching adolescents and young adults about mental disorders and providing information about their diagnosis and treatment can be an important step that can reduce the burden of health institutions because they are among the risk groups and mental disorders are frequently seen at these ages [8]. However, this situation carries more risk for Syrian immigrant university students with different cultures. Türkiye is a country that receives and sends immigrants and is a transit route due to its geography. Especially in recent years, there has been a lot of migration due to the civil war in the neighboring country of Syria [9]. The “immigration” phenomenon is a source of intense stress for the individual. Immigrant individuals are affected by the psychological difficulties brought about by loneliness, alienation, stigmatization, marginalization, being seen as worthless, regret, and communication problems due to not knowing the language of the host society, and develop psychological problems [10]. In the study conducted by Gül and Kaylı with Syrian university students, it was observed that they perceived social exclusion in dimensions such as economic, spatial, city-based, cultural, educational, and health [11]. While traumatic experiences and past losses of Syrian immigrant students before migration negatively affect their mental health, they are exposed to multiple traumas such as the difficulties they encounter in the country they arrive after migration. Pre-migration risk factors include the negative economic, educational, and occupational status in their own country, political situations, social support, roles, and the disruption of the social network [12]. A life and uncertainty between Syria and TURKIYE is seen in young people who migrated from Syria to TURKIYE. While young people act with their own culture on the one hand, they try to adapt to TURKIYE and Turkish culture on the other. Adaptation is not always a conscious process. The individual inevitably enters into an interaction with the environment they are in [13]. Individuals who have inadequate coping mechanisms for intense stressful situations experienced during the migration process are more prone to mental health problems such as depression, anxiety, adaptation problems, post-traumatic stress disorder, and schizophrenia [14]. Syrian university students experience difficulties in adapting to an environment in Turkiye that is different from their own culture. The feelings of loneliness, alienation, feeling worthless, the absence of relatives, and the regret of leaving them behind deeply affect individuals and cause them to experience intense stress [15]. In addition, the inadequacy of the social environment, the high expectations of their relatives about the regions they migrate to, and the disappointment of their expectations negatively affect their mental health [16]. When the immigrant individual is faced with such a difficulty and cannot cope with it, the conflicts shift to the psychological level, which is reflected in the body causing somatic disorders [17].

It has been observed that forced migration, which is a difficult and traumatic process, increases the mental problems experienced by Syrian students and negatively affects education life and social cohesion. They may have problems in receiving psychosocial support due to the language difficulties they experience, they may not be able to dismiss the trauma caused by migration, they may also be prone to mental illnesses and may not have enough information about seeking help [8]. From this point of view, it is important to increase the level of knowledge of Syrian immigrant university students, develop a positive perspective toward mental illnesses, and teach them the process of coping with difficulties. To recognize and treat mental problems before they become chronic, MHL is effective in the early diagnosis and treatment of students [18]. In addition, the fact that MHL is effective in increasing the level of knowledge of immigrant students, improving support-seeking behaviors, and reducing stigma ensures a positive development in their psychological well-being [19, 20].

The development of MHL levels of Syrian immigrant university students ensures a positive development of mental well-being. Delaying the treatment process in order not to receive a mental diagnosis and not to be stigmatized leads to a more pathological situation in students [21]. Increasing MHL and support-seeking behavior of Syrian immigrant students who are at risk for mental health will also lead to positive changes in attitudes and beliefs toward mental health students [22]. The results of the research conducted in a large sample in Switzerland determined that the MHL score predicted positive attitudes toward seeking help and the perceived need for treatment [23]. It is thought that the study will enable immigrant students to recognize mental problems at an early stage, apply for professional help in a short time, and receive appropriate health care in providing suggestions for planning interventions to increase MHL.

This study will also make an important scientific contribution to the literature in terms of increasing the mental health literacy of immigrant students, ensuring that they become aware of their mental problems at early stages, and developing a positive attitude toward receiving psychological first aid.

## Method

### Type of research

The study had a cross-sectional design. The population of the study consisted of 2000 Syrian immigrant students studying at Mardin Artuklu University. Power analysis was made for the study and journal format to determine the number of people to be included in the study with the G*Power 3.1 program. The effect size was taken as 0.15 as a medium level according to the multiple regression analysis determined by Cohen [24]. In order to exceed the 95% value in determining the power of the study, 107 people had to be reached at a significance level of 5% and an effect size of 0.15 (df = 2; F = 3,086). The study was completed with 326 Syrian immigrant students. The data were collected by the researchers from the students through Google Docs forms on the online platform between September and November 2024. The prevention of re-entry of data from the same device was ensured through IP and cookie control. Data increase was regularly monitored and the collection process was terminated when the data increase stopped for 1 month. The research was conducted on Syrian immigrant students attending different departments of a state university in the 2023–2024 academic year. Criteria for inclusion in the study: Being a student at Mardin Artuklu University, literate in Turkish, willing to participate in the research, and having adequate communication skills. Syrian students were included in the definition of immigrant students.

### Population and sample

The sample included Syrian immigrant students who passed the Turkish preparatory class and volunteered for the study. Syrian immigrant students were those who had settled in Mardin province because of forced immigration and were studying at university. Other foreign students were excluded. Other students, Arabs, and Assyrians, were excluded from the study. Mardin is preferred by Syrian immigrants because of its geographical and religious proximity and the kinship ties between them for centuries. The fact that the city where this study was conducted is a settlement that receives immigrants because of its location, students register for university, the number of immigrant Syrian students at the university is high and they need support is verbally stated by academic advisors. The difficulties Syrian immigrant university students experience in terms of success in classes, many of them have to work in ordinary jobs because of economic difficulties, and they cannot benefit from healthcare services to seek psychological support, on the other hand, employment problems and limitations in scholarship opportunities are obstacles to receiving psychosocial support.

## Data collection tools

A three-part questionnaire was used in the study.

### Descriptive questions for immigrant students

Questions related to the age, sex, disease status, and drug use of Syrian immigrant students were asked.

#### MHL Scale (MHLS)

The original form of the MHLS was developed by Jung et al. [25]. The Turkish validity-reliability study of the scale was performed by Göktaş et al. [26]. The MHLS consists of 22 items and three sub-dimensions [26]. There are 10 items in the Knowledge-Oriented MHL [26] (MHL-KO) sub-dimension, eight items in the Belief-Oriented MHL (MHL-BO) sub-dimension, and four items in the Source-Oriented MHL (MHL-SO) sub-dimension. The 18 items in the first two sub-dimensions of the scale are six-point Likert type. The four items in the MHL-KO sub-dimension are answered as “yes” and “no”. In the scale evaluation, one point is not taken from the “strongly agree”, “agree” and “yes” answers given to the items, and no points are taken from the other answers. Items 11, 12, 13, 14, 15, 16, 17, and 18 of the scale are reverse-coded. The total score that can be obtained from the scale varies between 0 and 22. Cronbach alpha value was found to be 0.89 for this study.

### Attitude Scale toward Receiving Professional Psychological Support-Short Form (ATSPPH-SF)

This form was used to evaluate the attitudes of the participants toward receiving psychological support. The original form of the ATSPPH-SF, the “Attitudes Scale toward Seeking Help from the Expert” was developed by Fischer and Turner [27]. It is a revised version of the Attitude Scale toward Receiving Psychological Support, which consists of 30 items and four subscales, adapted by Türküm [28], which aims to measure attitudes toward receiving psychological support [28]. The scale, which was reduced to 18 items after the revision, provides a five-point Likert-type measurement. The psychometric characteristics of the first form show that the scale is a valid and reliable measurement tool. ATSPPH-SF consists of 18 items with a 5-point Likert-type scale. The respondent is asked to read the statements in each item and mark 1 if they do not agree at all, 2 if they agree somewhat, 3 if they are undecided, 4 if they agree a lot, and 5 if they agree completely. The answers are scored in the same order from 1 to 5. Thus, the lowest score a respondent can get on the scale is 18, and the highest score is 90. A high score on the scale indicates a high positive attitude toward psychological support. The results of the factor analysis showed that the scale was collected in two factors. The first factor includes positive views about seeking psychological support, while the second factor includes negative opinions. The increase in the score obtained from the scale indicates that a positive attitude toward receiving professional psychological support is adopted. In the reliability study, the Cronbach alpha coefficient for the entire scale was 0.90. The internal consistency coefficients of the scale were found to be *r* = 0.87 for positive views on seeking psychological support, *r* = 0.70 for negative views on seeking psychological support, and *r* = 0 0.81 for the entire scale. Cronbach alpha value was found to be 0.87 for this study.

### Statistical analysis of data

The data obtained in the study were evaluated using the IBM SPSS Statistics for Windows, Version 22.0 statistical program (SPSS Inc., Chicago, IL, USA). Frequency and percentage analyses were used to determine the descriptive characteristics of the participants and mean and standard deviation statistics were used to examine the scale. To determine whether the research variables showed normal distribution, Kurtosis (kurtosis) and Skewness values were examined (Table 2). In the relevant literature, kurtosis skewness values of variables between + 1.5 and − 1.5 are accepted as normal distributions [29] and + 2.0 and − 2.0 [18]. It was determined that the variables showed normal distribution. Parametric methods were used in the analysis of the data. The differences between the ratios of categorical variables in independent groups were analyzed using Chi-square and Fisher exact tests. The relationships between the dimensions that determined the scale levels of the students were examined through Pearson correlation and linear regression analysis. Independent groups t-test, one-way analysis of variance (ANOVA), and post hoc (Tukey, LSD) analyses were used to examine the differences in scale levels according to the descriptive characteristics of the students. Cohen (d) and Eta square (η2) coefficients were used to calculate the effect size. The effect size indicates whether the difference between the groups is a large difference that can be considered significant. Cohen values are evaluated as 0.2: small; 0.5: medium; 0.8: large, and eta squared values are evaluated as 0.01: small; 0.06: medium; and 0.14: large [30].

### Ethical aspects of the research

The study was approved by Mardin Artuklu University’s Non-Interventional Clinical Research Ethics Committee. Ethical permission (11.06.2024, 2024/6–25) and institutional permission was obtained from Mardin Artuklu University Rectorate. Permission of the institution for the research was obtained from the Dean of the faculty and the university. For the scales used in the study, permission was obtained from the authors who conducted the validity and reliability study. After reading the informed consent form at the beginning of the research, the participants proceeded by selecting the “yes” option in the statement “I agree to be informed about the research and participate in the study.” Among the individuals who would participate in the study, those who approved the informed consent were included in the study. The study was conducted in line with the Helsinki Declaration and informed consent was obtained from the participants.

## Results

Table 1 shows the distribution of students according to their descriptive characteristics. Of the students included in the study, 62.3% were female, 50.3% had an income equal to their expenses, 91.1% did not have a diagnosed mental illness, and 88.7% did not have a diagnosed physical chronic illness. Similarly, the rate of students who said “no” in terms of the presence of a mental health diagnosis in the family (91.7%) and the use of psychiatric medication (91.1%) was quite high. Among the sources that students preferred to turn to when experiencing a psychological problem, the highest rate was stated as parents (37.1%). Friend (20.6%) and “nobody” (23.9%) were other notable answers. In terms of the idea of receiving professional psychological support, almost half of the students (48.2%) indicated this option as “yes”, but the rate of those who received professional help in the past remained at a lower level (12.9%). In terms of the average age, the average age of the students was determined to be 22,040 ± 3,075 and the age range was between 18 and 34 (Table 1).

**Table 1:** Descriptive characteristics

Groups	Frequency (*n*)	Percentage (%)
Sex		
Male	123	37.7
Female	203	62.3
Income Status		
Income less than expenses	162	49.7
Income equals expenses	164	50.3
Presence of Diagnosed Mental Health Illness		
Yes	29	8.9
No	297	91.1
Presence of Diagnosed Physical Chronic Disease		
Yes	37	11.3
No	289	88.7
Presence of Mental Health Diagnosis in the Family		
Yes	27	8.3
No	299	91.7
Use of Psychiatric Medication Prescribed by a Doctor		
Yes	29	8.9
No	297	91.1
Mother’s Education Status		
Primary School Graduate	84	25.8
Secondary School Graduate	77	23.6
High School Graduate	85	26.1
University Graduate	80	24.5
Father’s Education Status		
Primary School Graduate	48	14.7
Secondary School Graduate	56	17.2
High School Graduate	116	35.6
University Graduate	106	32.5
Presence of Unwanted Traumatic Experience		
Yes	89	27.3
No	237	72.7
The First Source of Reference in the Psychological Problem		
Mother and Father	121	37.1
Friend	67	20.6
Another Family Member	28	8.6
Doctor	15	4.6
Psychologist	17	5.2
Nobody	78	23.9
Thought of seeking professional psychological support		
Yes	157	48.2
No	169	51.8
Previous receipt of professional psychological support		
Yes	42	12.9
No	284	87.1
	Mean	SD
Age	22.04	3.07

Table 2 shows the descriptive statistics regarding the total mean scores of the scales used in the study.The mean scores of the 326 students who participated in the study regarding mental health literacy and attitudes toward seeking psychological support were evaluated. The mean overall Mental Health Literacy Total Score was found to be 11.804 ± 3.434. The level is evaluated as moderate. In the sub-dimensions, the Information-Oriented dimension mean was determined as 4.528 ± 3.202; the Belief-Oriented dimension mean was found to be 5.666 ± 2.211. The Source-Oriented dimension mean was 1.610 ± 1.539. These results show that the information-oriented dimension has a higher distribution among the sub-dimensions of mental health literacy. The lowest level is the Resource-oriented sub-dimension. The mean total score of Attitude toward Seeking Psychological Support was found to be 58.245 ± 8.131. When the sub-dimensions of this attitude were evaluated, the mean of the Positive Attitude dimension was found to be 39.497 ± 7.569, while the mean of the Negative Attitude dimension was 18.750 ± 4.295. Higher positive attitude scores indicate that students generally have a positive approach toward psychological support (Table 2). Kurtosis and Skewness values ​​were examined to determine whether the research variables showed a normal distribution (Table 2). In the relevant literature, the results regarding the kurtosis and skewness values ​​of the variables are accepted as normal distribution when they are between + 1.5 and − 1.5 [29], + 2.0 and − 2.0 [30].

**Table 2:** Mental health literacy and seeking psychological support

	*n*	Mean	SD	Min.	Max.	Kurtosis	Skewness	Alpha
MHL Total	326	11.80	3.43	1.00	20.00	−0.48	−0.05	0.86
Knowledge-oriented	326	4.53	3.20	0.00	10.00	−1.29	−0.02	0.84
Belief-oriented	326	5.67	2.21	0.00	8.00	0.245	−0.96	0.84
Resource-oriented	326	1.61	1.54	0.00	4.00	−1.34	0.40	0.84
Attitude toward Receiving Psychological Support Total	326	58.24	8.13	39.00	88.00	1.07	1.03	0.91
Positive Attitude	326	39.50	7.57	12.00	60.00	1,27	−0.09	0.90
Negative Attitude	326	18.75	4.29	6.00	30.00	0.60	0.17	0.88

### Pearson correlation analysis

Table 3 shows a positive and moderate correlation was found between the total score of MHL and the ATSPPH-SF total score (*r* = 0.514, *p* < 0.01). This finding shows that as individuals’ MHL levels increase, their attitudes toward receiving psychological support develop positively. There was no positive and moderate relationship between the knowledge-oriented dimension and the attitude toward receiving psychological support (*r* = 0.494, *p* < 0.01), a weak positive relationship between the Resource-oriented dimension and the total attitude score (*r* = 0.186, *p* < 0.01), and no significant relationship was found between the belief-oriented dimension and the psychological support attitude (*r*= −0.046, *p* > 0.05). A positive correlation was observed between positive attitude and MHL total score (*r* = 0.430, *p* < 0.01). In particular, a positive and strong relationship was found between the knowledge-oriented dimension and positive attitude (*r* = 0.525, *p* < 0.01). This result reveals that the increase in the level of knowledge of individuals about mental health strengthens positive attitudes. There was a low level of positive correlation between the Resource-oriented dimension and positive attitude (*r* = 0.153, *p* < 0.01). A negative correlation was found between the belief-oriented dimension and positive attitude (*r*= −0.198, *p* < 0.01). This finding suggests that an increase in belief-oriented thoughts may negatively affect positive attitudes toward seeking psychological support (Table 3).

**Table 3:** Inter-scale correlation relationship

		MHL Total	Knowledge-oriented	Belief-oriented	Resource-oriented
ATSPPH-SF Total	r	0.51	0.49	−0.05	0.19
	*p*	< 0.001	< 0.001	0.410	0.001
Positive Attitude	r	0.43	0.52	−0.19	0.15
	*p*	< 0.001	< 0.001	< 0.001	0.006
Negative Attitude	r	0.21	0.01	0.26	0.08
	*p*	< 0.001	0.859	< 0.001	0.140

Table 4 shows the results of the regression analysis showing the effect of MHL on the attitude towards seeking psychological support. When the values related to the explanatory nature of the model were examined, it was found that the rate of explaining the effect of the independent variable on MHL on the dependent variable, that is, receiving psychological support, was 26.2% (*R*²=0.262). The regression model was found to be significant (F = 116.392, *p* < 0.001) (Table 4).

**Table 4:** The effect of mental health literacy on seeking psychological support

Independent Variable	Non-Standardized Coefficients		Standardized Coefficients	t	*p*	95% Confidence Interval	
	B	SE	B			Lower	Upper
Constant	43.879	1.387		31.643	< 0.001	41.151	46.607
MHL Total	1.217	0.113	0.514	10.789	< 0.001	0.995	1.439

Dependent Variable = Attitude toward Receiving Psychological support Total, *R* = 0.514; R2 = 0.262; F = 116.392; *p* = 0.000; Durbin Watson Value = 2.015

Table 5 shows the results of the multiple regression analysis on the effect of the sub-dimensions of MHL on attitudes toward seeking psychological support. The overall explanatory rate of the model was found as 29.2% (*R*²=0.292), and the effect of independent variables on psychological assistance attitude was found to be significant (F = 45.589, *p* < 0.001). The Durbin-Watson value of the model, which indicates that there is no autocorrelation problem, was calculated as 1.981. Among the sub-dimensions, the knowledge-oriented dimension had the strongest effect (B = 1.496, β = 0.589, *p* < 0.001). Each unit increase in the knowledge-oriented dimension corresponded to a 1.496-unit increase in the psychological assistance attitude score. This finding clearly shows that the increase in the level of knowledge positively affects the attitudes of individuals to seek psychological support. The belief-based dimension had a significant and positive effect on the attitude toward psychological assistance (B = 0.918, β = 0.250, *p* < 0.001). A one-unit increase in the belief-oriented dimension provided a 0.918-unit increase in the psychological support attitude score. However, it was observed that the effect of this dimension was lower compared with the knowledge-oriented dimension. This finding shows that belief-based approaches may positively affect the attitude toward psychological support, but their effect is limited. The Resource-oriented dimension showed a significant but weaker effect on psychological assistance attitude (B = 0.531, β = 0.101, *p* = 0.037). A one-unit increase in the Resource-oriented dimension provided a 0.531-unit increase in the attitude score. This situation reveals that the effect of individuals’ knowledge and awareness of access to mental health services on attitude is limited but significant. These findings emphasize that knowledge-based MHL is the most important determinant of attitudes toward seeking psychological support. It is suggested that educational programs should prioritize knowledge growth and be supported by initiatives to reduce belief-based misunderstandings. In addition, it is thought that the level of knowledge of individuals about access to mental health services should be strengthened to increase the effect of the Resource-oriented dimension.

**Table 5:** Relationship between scales

Independent Variable	Non-Standardized Coefficients		Standardized Coefficients	t	*p*	95% Confidence Interval	
	B	SE	B			Upper	Lower
Constant	45.417	1.590		28.558	< 0.001	42.288	48.546
Knowledge-oriented	1.496	0.136	0.589	10.994	**<** 0.001	1.228	1.764
Belief-oriented	0.918	0.195	0.250	4.698	**<** 0.001	0.533	1.302
Resource-oriented	0.531	0.254	0.101	2.091	0.037	0.031	1.031

Dependent Variable = Attitude toward Seeking Psychological Support, R = 0.546; R2 = 0.292; F = 45.589; *p* ≤ 0.0001; Durbin Watson Value = 1.981

Table 6 shows the distribution of the scales according to variables. The scores of the students who did not use medication were significantly higher than those who used medication, and the MHL total scores of the students who had the idea of seeking psychological support (12.66 ± 3.52) were significantly higher than those who did not (11.01 ± 3.16) (*p* < 0.001) (Table 6). In terms of sex, it was determined that the total attitude scores of female students toward seeking psychological support (59.49 ± 9.06) were significantly higher than those of male students (56.19 ± 5.77) (*p* < 0.001). When the income status was examined, the total scores of psychological assistance attitudes (59.12 ± 8.72) of the students whose income was equal to their expenses were found to be higher than those whose income was less than their expenses (57.36 ± 7.41) (*p* = 0.050). The total scores of the students who used psychiatric drugs (54.76 ± 6.33) were found to be lower than those who did not (*p* = 0.015). Students who had the idea of receiving psychological support had significantly higher total scores compared with those who did consider receiving help (*p* < 0.001). This shows that the general attitudes of individuals who tend to receive help are more positive (Table 6).

**Table 6:** Distribution of scales according to variables

Demographic Characteristics	*n*	MHL Total	ATSPPH-SF Total
Sex		Mean ± SD	Mean ± SD
Male	123	10.65 ± 3.39	56.19 ± 5.77
Female	203	12.50 ± 3.28	59.49 ± 9.06
t=		−4.882	−3.609
p=		< 0.001	< 0.001
Income less than expenses	162	12.06 ± 3.26	57.358 ± 7.41
Income equal to expenses	164	11.55 ± 3.59	59.122 ± 8.72
t=		1.318	−1.967
p=		0.189	0.050
Presence of Diagnosed Physical Chronic Disease Mental Health Illness			
Yes	29	10.86 ± 3.36	55.55 ± 9.78
No	297	11.90 ± 3.43	58.51 ± 7.92
t=		−1.550	−1.876
p=		0.122	0.062
Presence of Diagnosed Chronic Disease			
Yes	37	12.38 ± 2.96	59.00 ± 10.24
No	289	11.73 ± 3.49	58.15 ± 7.84
t=		1.081	0.599
p=		0.225	0.550
Presence of Mental Health Diagnosis in the Family			
Yes	27	11.78 ± 3.73	56.852 ± 10.77
No	299	11.81 ± 3.41	58.371 ± 7.86
t=		−0.041	−0.930
p=		0.967	0.353
Use of Psychiatric Medication Prescribed by a Doctor			
Yes	29	10.45 ± 3.45	54.74 ± 6.33
No	297	11.94 ± 3.41	58.59 ± 8.21
t=		−2.240	−2.438
p=		0.026	0.015
Presence of Unwanted Traumatic Experience			
Yes	89	12.169 ± 3.27	57.751 ± 7.65
No	237	11.667 ± 3.49	58.099 ± 7.98
t=		1.176	1.797
p=		0.240	0.073
The First Source of Reference in the Psychological Problem			
Mother and Father	121	11.68 ± 3.40	55.60 ± 5.72
Friend	67	11.75 ± 3.87	59.67 ± 9.32
Another Family Member	28	12.50 ± 3.02	58.79 ± 6.82
Physician	15	11.40 ± 3.87	55.60 ± 5.72
Psychologist	17	13.35 ± 3.39	61.12 ± 10.66
Nobody	78	11.54 ± 3.13	56.94 ± 7.26
F=		1.094	1.606
p=		0.363	0.158
PostHoc=			
Thought of Seeking Professional Psychological Support			
Yes	157	12.66 ± 3.52	59.50 ± 9.50
No	169	11.01 ± 3.16	56.651 ± 6.37
t=		4.477	3.747
p=		< 0.001	< 0.001
Previous Receipt of Professional Psychological support			
Yes	42	12.90 ± 3.85	58.786 ± 10.522
No	284	11.64 ± 3.34	58.17 ± 7.73
t=		2.240	0.461
p=		0.026	0.645

## Discussion

The findings reveal that immigrant students’ attitudes toward psychological assistance differ according to various demographic characteristics and life experiences, showing some important parallels and differences when compared with the literature. It was found that the psychological assistance attitudes of immigrant female students were more positive than immigrant male students (*p* < 0.001) (Table 6). This is also supported in the literature; it has been stated that female students’ support-seeking behaviors are generally more positive, and their social support and emotional awareness affect this attitude [31–34]. In our study, it is thought that male students’ low MHL and psychological support-seeking attitudes may be related to gender roles. The expectation of men in society to be stronger, tougher, and exhibit behaviors that do not show their emotions, unlike women, and the fact that women seek more information and attach more importance to mental health may have affected this situation [35–37].

Income status made a significant difference in immigrant students’ attitudes toward psychological assistance; individuals whose income was equal to their expenses had higher positive attitude scores (*p* = 0.036). This coincides with findings that economic concerns can negatively affect support-seeking behaviors [38]. It can be said that individuals with lower income levels may see psychological support as a financial burden and therefore their attitudes may be negatively affected. On the other hand, although there was no significant relationship between the income status of Syrian immigrant students and MHL (*p* > 0.05), it was observed that individuals with low-income status scored higher in the resource awareness dimension. This can be attributed to studies showing that economic hardship encourages individuals to seek alternative health sources [39]. In this context, it is possible to argue that Syrian immigrant students are forced to work in unqualified jobs with no insurance and low wages, which is not reflected in the literature. Students also benefit sufficiently from some scholarships given by universities.

It was found that the positive attitude scores of the students with a mental diagnosis were lower (*p* = 0.001), but the negative attitude scores were not higher. This suggests that the support-seeking behaviors of diagnosed individuals may have been influenced by stigma or past experiences [40]. Similarly, the attitudes of individuals using psychiatric drugs were more negative (*p* < 0.001), suggesting that the obstacles faced by these individuals in the process of seeking help were reflected in their psychological attitudes. It was determined that the general attitudes of those who had the idea of receiving psychological support from Syrian immigrant students were more positive (*p* < 0.001). This result is consistent with the literature, which states that individuals’ support-seeking behaviors are often linked to mindfulness and positive attitudes [39]. For example, in a study sample conducted with Malaysian students, it was stated that the reason for students’ negative attitudes toward receiving psychological support was their negative beliefs about receiving psychological support [41]. In the study, it was found that Syrian immigrant students with a mental diagnosis scored lower in the knowledge-oriented dimension (*p* = 0.002), but did not differ significantly in other dimensions. This finding is in line with findings by Jorm that individuals with mental health problems sometimes have difficulty accessing information and that support-seeking behaviors may be affected [42]. In the compilation study conducted on the problems experienced by Syrian students under temporary protection, communication problems, individual problems, academic problems, and family-related problems were determined. It has been observed that forced migration, which is a difficult and traumatic process, increases the mental problems experienced by Syrian students and negatively affects education life and social cohesion [43]. Based on the results of 192 epidemiological studies, which are large-scale meta-analyses of the age of onset of mental disorders and mental illnesses worldwide, the rate of individuals with the onset of any mental disorder before the ages of 14, 18, and 25 was reported as 34.6%, 48.4%, and 62.5%, respectively. In the present study, the age of onset of mental illnesses was found to be similar to that of immigrant students according to their age ranges [7].

The attitude toward seeking psychological support and MHL levels may be effective in coping with challenging situations they encounter in young adulthood. It was observed that Syrian immigrant students who had a psychological diagnosis in their family had higher scores in the Resource-oriented dimension (*p* = 0.043). In addition, Syrian immigrant students using psychiatric drugs have a lower score in the knowledge-oriented dimension (*p* < 0.001), which may indicate that the information needs of students with experience in receiving help are more pronounced. Clement et al. (2015) stated that family history and previous experiences could increase MHL [44]. There was no significant difference between individuals who experienced traumatic experiences and those who did not (*p* > 0.05). However, the fact that the first source of reference is parents or friends can affect MHL. In particular, the high general literacy scores of the students who applied to psychologists (*p* = 0.007) emphasized the importance of professional support for mental health awareness. It was found that the MHL scores of the students who had the idea of seeking professional help were significantly higher than those who did not (*p* < 0.001). This result supports the findings of Rickwood et al. (2007) who stated that support-seeking behaviors were linked to knowledge levels and awareness [31]. Awareness programs aimed at increasing the mental health literacy of Syrian immigrant university students might help increase their attitudes toward seeking psychological support. It was reported in the literature that Syrian immigrant university students have an increased risk of post-traumatic stress disorder, anxiety disorders, self-alienation, schizophrenia, somatization disorder, and psychotic features as a result of not receiving psychological support after the war [13]. In a study conducted to examine the difficulties faced by Syrian immigrant university students in Turkiye, it was found that they did not have a certain sense of belonging, social adaptation received education in return for tuition fees, they were concerned about not being able to enter the workforce after graduation and being able to travel by obtaining a permit [45]. The inability to cope with these difficulties has negative impacts on mental health over time and may prevent seeking psychological support.

The MHL sub-dimension clearly shows that the increase in the level of knowledge positively affects the attitudes of individuals to seek psychological support. The belief-based dimension had a significant and positive effect on the attitude toward psychological assistance (B = 0.918, β = 0.250, *p* < 0.001). Belief emphasizes that information-oriented MHL is the most important determinant of attitudes toward seeking psychological support. Students with high MHL can identify mental problems and disorders and identify appropriate sources of support and treatment. MHL training may be needed for students to improve their mental health and have a positive attitude toward seeking psychological support [46]. In a study of college students, MHL supports positive psychological support-seeking attitudes, individuals’ ability to seek help, and mental health [47].

### Limitations of the study

It was not possible to receive full feedback from all classes and departments at the desired level because participation in the study was expected to be voluntary. However, the minimum number of students expected to be reached was reached.

## Conclusion

It shows that the MHL of Syrian immigrant students differs according to sex, income level, and individual experiences. It is noteworthy that MHL is higher, especially for women and students who are considering seeking professional help and those who receive psychological support. In our study, there is a significant relationship between belief-oriented MHL and the attitude toward receiving psychological support, and it was determined that the belief systems of individuals affected their positive attitudes toward receiving psychological support. Factors such as sex, income status, and psychiatric drug use can significantly affect support-seeking attitudes. These findings have been important in highlighting the need for personalized intervention programs to improve individuals’ support-seeking behaviors. It is recommended that training programs focus on increasing the level of knowledge, clearing up belief-based misconceptions, and strengthening resource awareness. In addition, it is thought that the level of knowledge of individuals about access to mental health services should be strengthened to increase the effect of the Resource-oriented dimension. It was not possible to examine the status of mental health literacy or psychological support seeking in which diagnoses because the study did not differentiate between mental diagnoses. At this point, it might be recommended to conduct studies on mental health literacy and attitudes toward seeking psychological support in diagnosed immigrant individuals. Another suggestion is that since the status of immigrant students participating in the study was not questioned as a forced or voluntary migration, it is recommended to examine the differences in future studies. In this context, it is recommended that activities be organized to ensure the integration of Syrian students and their participation in social activities at universities.

### Inclusion criteria

Those who met the criteria of being a Syrian immigrant undergraduate student at a state university and volunteering to participate in the study with a mobile application were included.

## Supplementary Information

Supplementary Material 1.

## Data Availability

The data that supports the findings of this study is available from the corresponding author upon reasonable request.
