# Supplementary material for: The relationship between mental health literacy and psychological support-seeking attitudes in Syrian immigrant students
Source: BMC Public Health. 2025 Sep 30;25:3223. doi: 10.1186/s12889-025-23816-8 (PMC12486662; doi:10.1186/s12889-025-23816-8)
Supplement: Supplementary file 1 — Supplementary Material 1. [file 12889_2025_23816_MOESM1_ESM.pdf]

akademik makale
info@akademikmakale.net
www.akademikmakale.net

May 22, 2025

# To Whom It May Concern

Manuscript Title:
"The Relationship between Mental Health Literacy and Psychological Support Seeking Attitudes in Syrian Immigrant Students"

The proofreading and/or editing of the manuscript mentioned above were performed by a native speaker. We hereby confirm that the following issues have been corrected: grammar, spelling, punctuation, sentence structure, phrasing and style, and confirm that any language errors in this article have been rectified.

Yours Sincerely,

AKADEMİK TERCÜME
Mustafa Coşkun ZEREN
Karşıyaka Mh. Gekşi Cd.7.Sk.
Cep:0542 609 10 30 TOKAT
T.C.No: 5908 0409 678

Disclaimer: *The research or the authors' intentions remain unaltered during the proofreading / editing process. This document should be ready for publication. The author and journal is free to accept or reject our editing but we are not responsible for any changes made following our proofreading and editing.*
